# Exploring associations between metabolites and gene transcripts of common bean (*Phaseolus vulgaris* L.) in response to rust (*Uromyces appendiculatus*) infection

**DOI:** 10.1186/s12870-025-06584-w

**Published:** 2025-05-01

**Authors:** Penny Makhumbila, Molemi Rauwane, Hangwani Muedi, Ntakadzeni E. Madala, Sandiswa Figlan

**Affiliations:** 1 Department of Agriculture and Animal Health, School of Agriculture and Life Sciences, College of Agriculture and Environmental Sciences, University of South Africa, 28 Pioneer Ave, Florida Park, Roodepoort, 1709 South Africa; 2 Department of Botany, Nelson Mandela University, South Campus, University Way, Summerstrand, Port Elizabeth, 6001 South Africa; 3 Research Support Services, North-West Provincial Department of Agriculture and Rural Development, 114 Chris Hani Street, Potchefstroom, 2531 South Africa; 4 Department of Biochemistry, School of Mathematical and Natural Sciences, University of Venda, University Rd, Thohoyandou, 0950 South Africa

**Keywords:** Common bean rust, Transcriptomics, Metabolomics, Omics integration

## Abstract

Common bean (*Phaseolus vulgaris* L.) faces escalating challenges resulting from the increasing prevalence of fungal pathogens such as rust caused by *Uromyces appendiculatus*, threatening yields and quality of the crop. Understanding *P. vulgaris*’ disease response mechanisms is pivotal for the crop’s resilience and food security. Current scientific understanding of underlying molecular mechanisms of *P. vulgaris* to *U. appendiculatus* is limited, particularly with respect to specialised molecular data, including metabolite profiles and gene expression. There is a significant knowledge gap in explicating precise metabolomic and transcriptional changes that occur in *P. vulgaris* upon interaction with *U. appendiculatus*, which limits strategies aimed at enhancing pathogen resistance. In this study, biological stress response strategies of common bean to the rust pathogen were elucidated through a combined metabolomic and transcriptomic profiling approach. Our findings revealed that *U. appendiculatus* triggered diverse levels of 30 known metabolites, primarily flavonoids, lipids, nucleosides, and phenylpropanoids among others. Transcriptome sequencing detected over 3000 differentially expressed genes, including multiple transcription factor families such as heat shock proteins (HSPs), cytochrome P450 monooxygenases (CYP), terpene synthases and WRKY transcription factors (TFs) among others. Integrative metabolome and transcriptome analysis showed that rust infection enriched metabolomic pathways, biosynthesis of secondary metabolites, protein processing in the endoplasmic reticulum, and purine metabolism among others. The metabolome and transcriptome integration approach employed in this study provides insights on molecular mechanisms underlying *U. appendiculatus* response in *P. vulgaris*’ key developmental stages.

**Supplementary Information:**

The online version contains supplementary material available at 10.1186/s12870-025-06584-w.

## Introduction

Autoecious and macrocyclic pathogen, *Uromyces appendiculatus* (Pers.:Pers) Unger causes significant yield losses in common bean (*Phaseolus vulgaris* L.), one of the world’s vital legumes [1, 2]. Increased occurrence of pathogens such as *U. appendiculatus* in common bean production areas causes major yield losses, impacting food security [2, 3]. On the host plant, the formation of pycnia, aecia, and aeciospores gives rise to urediniospores, which rapidly multiply, ultimately triggering an epidemic. Later in the season, the pathogen completes its sexual lifecycle by producing basidiospores from teliospores, which facilitate overwintering and survival until the next growing season [4]. Moreover, the pathogen is highly variable and has several races that are virulent to common bean varieties with either one or a combination of rust resistance genes, thus complicating host plant resistance dynamics [2].

Physiologically, common bean utilises resistance via its rapid detection and response mechanisms, including a hypersensitive response (HR) that triggers cell death to limit pathogen spread [5]. Cell wall re-enforcement through lignification, callose deposition and accumulation of phenolic compounds are also strategies used by the plant to inhibit pathogen penetration [6]. At molecular level, broad spectrum resistance to the pathogen has been mapped using RAPD (Random Amplified Polymorphic DNA) and SCAR (Sequence characterized amplified region) molecular markers linked to the Mesoamerican (*Ur-3*, *Ur-5*, *Ur-7*, *Ur-11* and *Ur-14*) and Andean gene pools (*Ur-4*, *Ur-6*, *Ur-9*, *Ur-12* and *Ur-13*) located on chromosomes *Pv01*, *Pv04*, *Pv06*, *Pv07*, *Pv08* and *Pv11* [7, 8]. These genes encode NLR (nucleotide-binding leucine-rich repeat) proteins that can recognise pathogen effectors and initiate immune signalling [9]. Whilst studies on RAPD and SCAR markers have advanced molecular breeding for *U. apendiculatus* resistance, their PCR (polymerase chain reaction) dependency and amplification requirements present reproducibility challenges [10, 11]. In common bean production areas, *U. appendiculatus* has been controlled using fungicidal chemicals [12]. Furthermore, the incorporation of cultural practices such as crop rotation, deep ploughing and adjusting the planting date among others have been found to mitigate the impacts of the pathogen [13]. Interestingly, in Ethiopia, farmers were found to only rely on weeding to manage the spread of *U.* appendiculatus, due to limited knowledge of the pathogen and its management strategies [14]. Beyond this, the use of biological methods such as *Trichoderma spp.* has been explored [15]. However, the negative impacts of chemicals have been widely discussed [16, 17]. On the other hand, the use of cultural, biological and even integrated management practices does not provide full efficacy in pathogen management [18]. Therefore, the development of resistant cultivars can provide a steadfast solution to the production of *P. vulgaris* in *U. appendiculatus* prevalent areas. Metabolomics, a key discipline within omics science, focuses on extensive quantification of metabolites/compounds that are present in specific tissues and further aids in unravelling underlying regulatory systems that are linked to phenotypes under stress conditions [19]. Previously, metabolomic studies revealed that *U. appendiculatus* induced regulation of flavonoids, terpenoids and alkaloids among others [20]. Transcriptomics, another key omics field, captures transcripts, thus enabling the identification of genes and their roles under specific conditions [19]. To date, there is limited information on transcriptome profiling of common bean’s response to *U. appendiculatus,* resulting in a lag in common bean breeding initiatives for rust resistance when compared to other major crops.

Conversely, integrated metabolomics and transcriptomics of common bean in response to *U. appendiculatus* remains poorly explored. In-depth understanding of specialised metabolite and gene response to *U. appendiculatus* virulent races commonly found in the crop’s production areas can aid in the development of resistant varieties. Furthermore, the deployment of varieties with broad-spectrum resistance to several virulent races of the pathogen will ensure sustainable crop production, while also meeting food security goals. Using metabolomics and transcriptomics, we analysed specialised defence mechanisms of two varieties previously used as parental lines in breeding programmes, namely Golden Gate Wax and Teebus-RR-1 in response to *U. appendiculatus* race 31 − 1.

## Materials and methods

### Plant material, growth conditions and treatments

Prior to conducting the study, several available varieties were evaluated for their response to *U. appendiculatus* race 31 − 1 (Fig. S1). Evident to their common use as parental lines in breeding programmes [21], two varieties, Golden Gate Wax (susceptible variety harbouring *Ur-3* gene) and Teebus-RR-1 (resistant variety harbouring *Ur-3* and *Ur-6* genes) were selected for further evaluation. Both varieties were provided by the Agricultural Research Council (ARC - Grain Crops Institute, Potchefstroom, South Africa). Seeds were surface sterilised with a 50% bleach solution, rinsed with double distilled water and pre-germinated in Petri dishes (Lasec, South Africa). The pre-germinated seeds were grown in 9 cm pots using 30 dm^3^ sterile seedling soil (Cultera, South Africa) and covered with vermiculite. The seedlings were fertilised with multi-feed soluble NPK (19:8:16) fertiliser containing other macro- and micro- nutrients (Efekto, South Africa).

South Africa’s prevalent and virulent strain race 31 − 1 of *U. appendiculatus* previously isolated, purified and characterised by the ARC-GCI (South Africa) was obtained (stored under − 80 °C ultra-freezer conditions) for inoculation on the first trifoliate leaves of seedlings. Re-hydration of urediniospores was done by placing open cryotubes in a glass beaker with warm water, vermiculite, sealed with sterile cling wrap and left at room temperature (± 18 °C) for 12 h. To prepare the inoculum solution, 60 mg of spores, 50 ml of a Tween 20 and distilled water solution (5 drops/L; P1379, Sigma-Aldrich, Merck, United States) was prepared [22]. The solution was vigorously shaken, concentration of inoculum adjusted 2.5 × 10^4^ using a haemocytometer prior to pressure spraying (30 kPA) approximately + 0.5 ml of the suspension on the underside leaf. The control experiment was subjected to the same conditions as *U. appendiculatus* treated plants, however, inoculated with distilled water. Upon the inoculum drying, the seedlings were placed in a humidity chamber with 95–100% distilled water relative humidity for 48 h, at temperatures of ± 18 – ± 20 °C. After the 48 h of incubation, plants were moved to separate greenhouse compartments based on treatment, with 28/14°C day/night temperatures and 75% relative humidity. Furthermore, two days after removal from incubation chamber, the plants were transferred into 50 L grow bags with 5 plants per bag in a randomized complete block design [23].

### Phenotypic evaluations, metabolome and transcriptomic profiling

Evaluation of plants was done throughout the study duration, with specific interest in the plant’s response at 14- and 21- days post infection (dpi) with *U. appendiculatus*. In the duration of the experiment, both varieties were phenotypically evaluated and scored for disease severity [24, 25]. Three replicates of leaf samples were harvested from all treatments at 14- and 21-dpi prior to metabolomic and transcriptomic profiling. Samples were snap-frozen in liquid Nitrogen (N) and later kept in a -80 °C ultra-freezer.

Samples from both race 31 − 1 infected plants and mock inoculated/control plants for both varieties evaluated at 14- and 21-dpi were ground to powder in liquid N using mortar and pestle [26]. Prior to metabolite profiling, extraction was performed following established protocols and analysed using a liquid chromatography-quadrupole time-of flights tandem mass spectrometry instrument (LCMS-9030 qTOF, Shimadzu Corporation, Kyoto, Japan) [27]. For transcriptome profiling, a ZymoBIOMICS (Zymo Research, USA) kit and RNeasy (Qiagen, USA) kit were used for total RNA extraction and cleanup for all the 24 samples. Furthermore, the concentration of RNA samples was evaluated using Qubit^®^ fluorometer (ThemoFisher Scientific, USA). The total RNA extracts of samples was passed for quality if the RNA integrity number (RIN) was ≥ 7.5 [28]. Library preparation and RNA sequencing was done using an MGI DNBSEQ-G400 (MGI Tech, China) instrument at the Agricultural Research Council – Biotechnology Platform (South Africa).

### Data analysis

#### Metabolomic data processing and mining

The obtained DDA MS/MS data was processed using Mzmine3 (v4.2.40) [29] using the HPLC-qTOF-DDA processing mode for chromatogram smoothing, ionisation stabilisation across all samples. Parameters set in Mzmine3 included retention time range: 0.50–15.0 min, alignment, peak detection (minimum feature height: 1.0E3, deconvolution and noise detection (MS1: 5.0E2 and MS2.MSn: 1.0E2). The MZmine3-processed data were annotated using GNPS [30], while SIRIUS 6.0.4 was used for chemical class prediction and structural annotation (confidence level 2) [31, 32]. Databases KEGG (Kyoto Encyclopedia of Genes and Genomes), PubChem, SuperNatural, MetaCyc, Chemical Entities of Biological Interest (ChEBI), COCONUT and KNApSAcK were used for feature annotation and validation [31]. In addition, MetaboAnalyst (v6.0 [33] was used for statistical analysis (Fig. S2). The data was further analysed in SIMCA v18 (Umetrics, Sweden) to observe the general overview of the data and outlier presence using unsupervised Principal Component Analysis (PCA) model. Additionally, orthogonal partial least squares (OPLS) model was applied to the data to observe treatment differences. The metabolomic raw data was submitted to MetaboLights repository with accession number MTBLS6972.

#### Transcriptome data analysis, validation of DEGs and integrated analysis

**Fig. 1:**
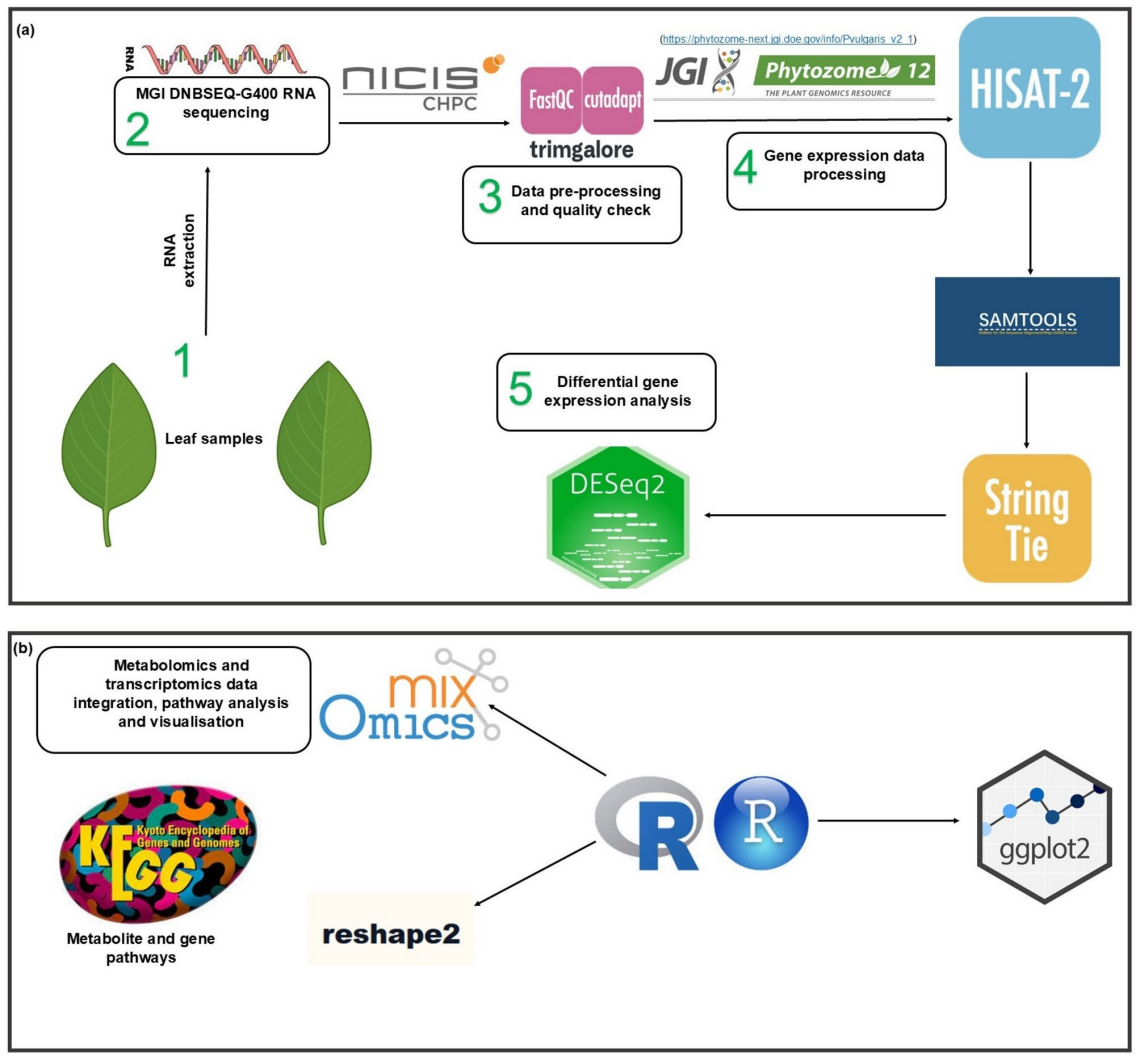
Transcriptomics and metabolomics integration workflow (**a**) A schematic representation of transcriptome data collection, processing and statistical analysis. Consequent to leaf sampling (step 1), gene expression data was acquired (step2) and subjected to quality check (step 3), genome alignment and sorting (step 4) and DEG analysis (step 5). (**b**) Metabolite and gene transcript integration including pathway analysis

Raw sequence data was assessed for quality in FastQC v0.11.5 and adaptors together with ambiguous sequences trimmed using TrimGalore v0.6.5. *Phaseolus vulgaris* v2.1 (https://phytozome-next.jgi.doe.gov/info/Pvulgaris_v2_1) from the Plant Genomics Resource (JGI) was used as a reference genome for alignment and assembly in HISAT2 v2.0.6 [34]. Furthermore, the data was sorted in Samtools v1.9 [35] before the assembling of sorted RNA-Seq reads into potential transcripts in StringTie V2.2.1 [36]. DESeq2 v1.42.1 was used to analyse the gene expression matrix from StringTie to obtain differentially expressed genes (DEGs) with P-adjust < 0.05 and|log2FC| ≥1 [37]. The workflow/steps (Fig. 1a) and parameters used in the data analysis of this study have been previously described [38]. The sequence raw data was submitted into NCBI Sequence Read Archive (SRA) with bio project ID: PRJNA1061833.

Among the observed DEGs, only eight genes were common across all treatments (Table S2). From this set, five (5) genes were selected for downstream quantitative real-time (qRT) PCR analysis to ensure consistency and reliability. This approach of using common genes across treatments is a well-established strategy, as it minimises variability and enhances robustness of gene expression studies [39]. Primers used for qRT-PCR analysis were designed using NCBI’s primer blast (https://www.ncbi.nlm.nih.gov/tools/primer-blast/; Table S1). Conditions from primers included amplicon lengths of < 200 bp, 50–60 ºC melting temperatures and 40–60% G content. Reverse transcription of RNA (RT) was done using Takara’s PrimeScript 1st strand cDNA synthesis kit (Takara Bio, Europe, France). The quantification of complementary DNA (DNA) was done using Qubit ^®^ fluorometer with concentrations of 50ng prepared for each sample. Inqaba Biotec (Inqaba Biotechnical Industries, South Africa) conducted qRT-PCR analysis using the CFX 96 Real-Time PCR System (Bio-Rad, Hercules, CA). The reference gene used to normalise the Ct values was Actin [40]. The ^ΔΔ^Ct method was used to calculate the relative expression of genes [41].

Annotated metabolites and DEGs common across treatments (14- and 21-dpi; Table S2, S3 and S4) were used for pathway enrichment analysis in KEGG [42]. Furthermore, the Data Integration Analysis for Biomarker discovery using Latent cOmponents (DIABLO) mixOmics model [43] was applied in R to integrate metabolomics and transcriptomics data (Fig. 1b). The DIABLO model uses a multivariate approach (canonical correlation analysis: CCA) to extract latent components that capture underlying layers of omics data to discover molecular signatures [44].

## Results

### Phenotypic variations at time points of evaluation

**Fig. 2:**
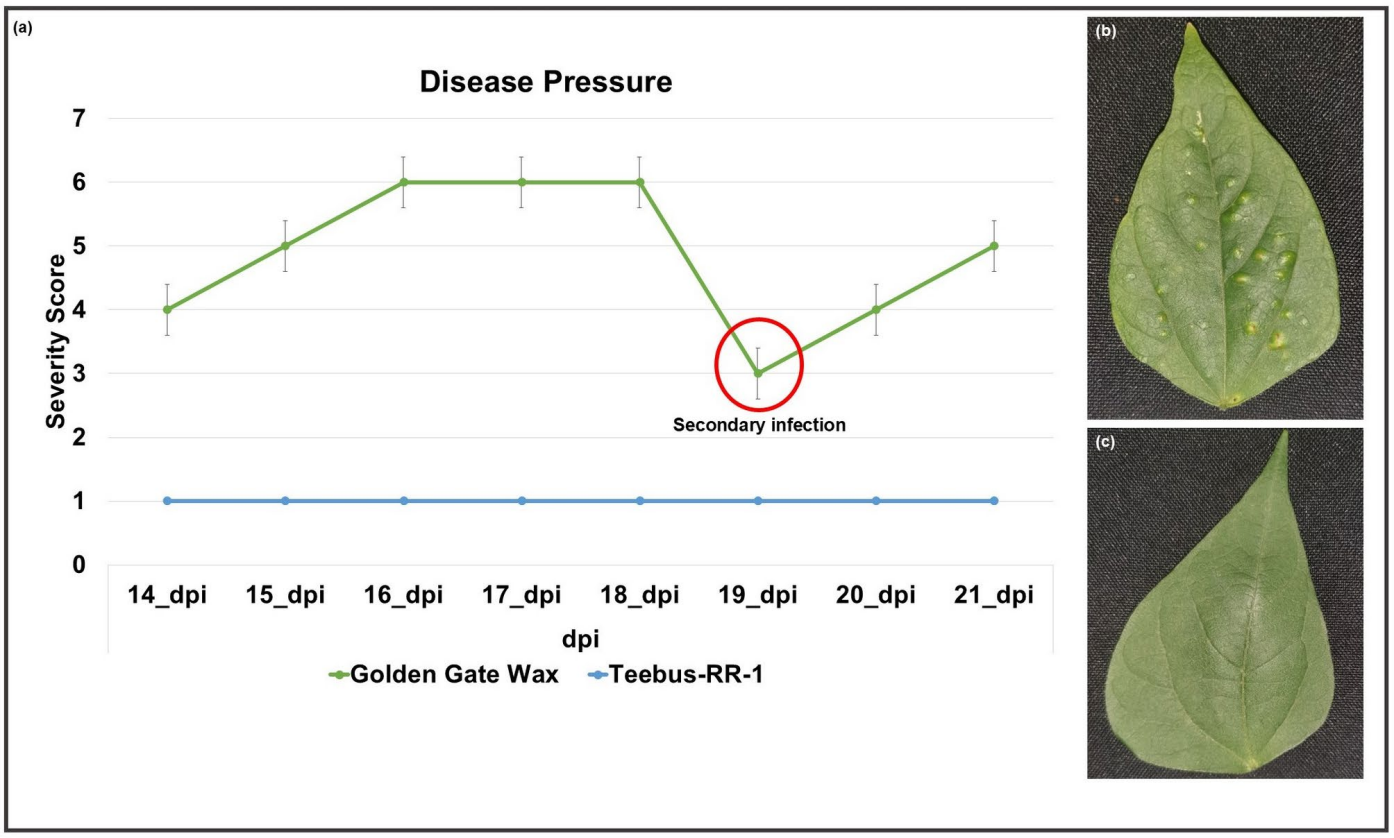
Disease score status and phenotypic observations at 21-dpi. (**a**) Severity score graph. (**b**) Leaves highlighting phenotypic observations between Golden Gate Wax and Teebus-RR-1 from 14- to 21-dpi. The red circle indicates secondary infection stage by the pathogen in Golden Gate Wax

To observe phenotypic variations between varieties, leaves of both Golden Gate Wax and Teebus-RR-1 were scored for disease severity, observing the pustule sizes [24]. Golden Gate Wax had increased disease pressure from 14-dpi and at 21-dpi. Interestingly, although not a sampling point, at 19-dpi, Golden Gate Wax showed signs of secondary infection (Fig. 2a, b). Meanwhile Teebus-RR-1 exhibited no symptoms of the pathogen at 14 and 21-dpi (Fig. 2a, c).

### Metabolite regulation patterns of *P. vulgaris* in response to *U. appendiculatus*

**Fig. 3:**
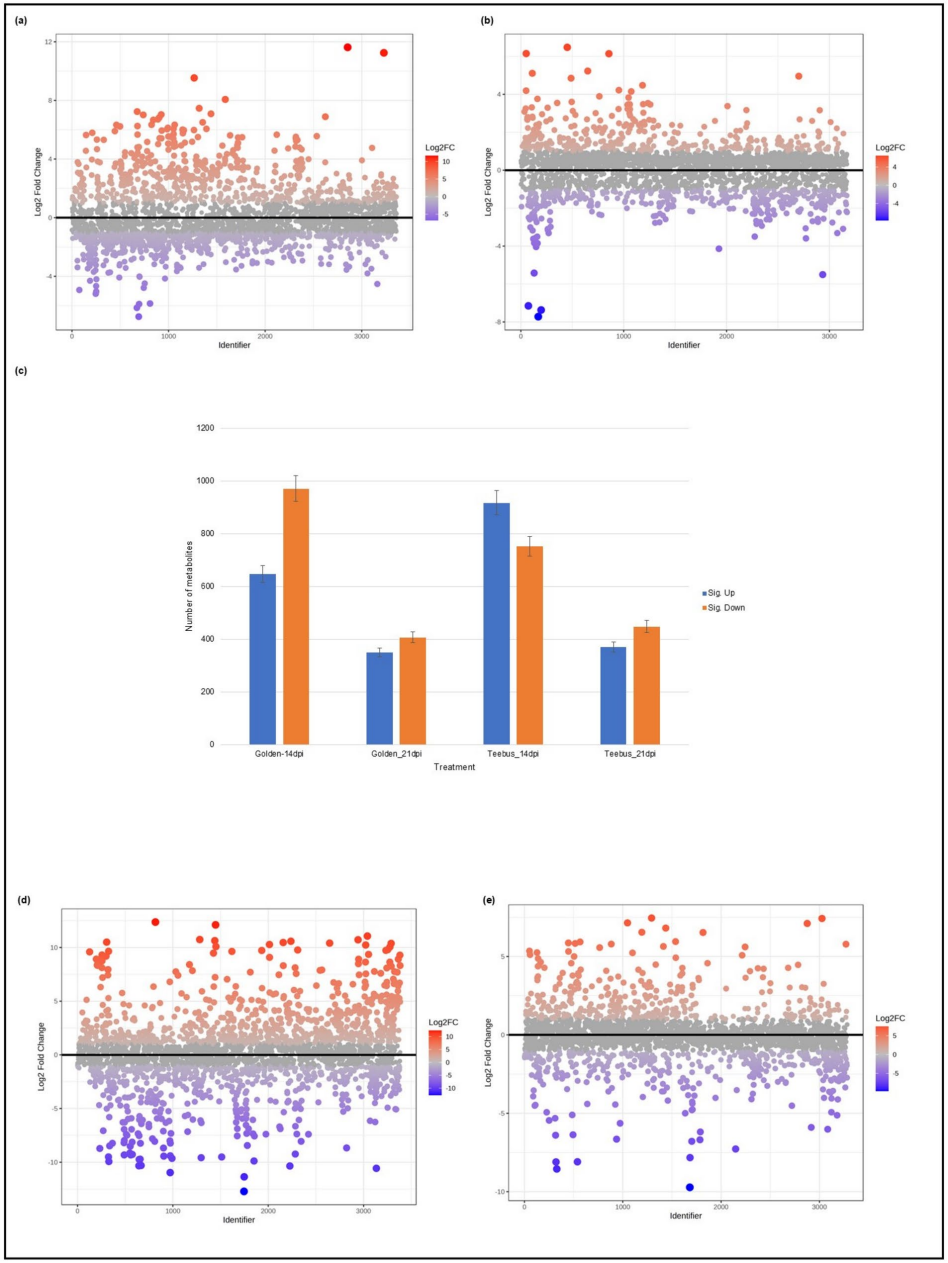
Volcano plots showing log2FC metabolite variations among leaf samples. (**a**) Golden Gate Wax at 14-dpi, (**b**) Golden Gate Wax 21-dpi, (**c**) Overall DEG regulation patterns between varieties at 14- and 21-dpi (**d**) Teebus-RR-1 at 21-dpi. Comparisons of race 31 − 1 infected samples to control/mock inoculated samples. Differential expression of metabolites with *P*-value ≤ 0.05 and a log2FoldChange (FC) ≥ 1 considered to be significantly different

Metabolites generated by the LCMS-9030 qTOF instrument were regulated differentially between Golden Gate Wax and Teebus-RR-1 at both time points. At 14-dpi, Golden Gate Wax up-regulated > 900 metabolites, while down-regulating > 600 metabolites. At 21-dpi, the proportion of up- and down-regulated metabolites was comparable in Golden Gate Wax (> 300). Furthermore, the up- and down-regulated metabolites were between 10 and − 5 log2FC at 14dpi (Fig. 3a and c). At 21-dpi, Golden Gate Wax showed up- and down-regulation of ± 4 log2FC (Fig. 3b). Teebus-RR-1, on the other hand, had a higher proportion of down-regulated metabolites (> 900), while > 600 metabolites were up-regulated at 14-dpi. At 21-dpi, > 300 metabolites were up- and down regulated by Teebus-RR-1 (Fig. 3c). Teebus-RR-1 elevated metabolite levels by approximately 10 log2FC at 14 dpi and 5 log2FC at 21 dpi (Fig. 3d and e).

### *U. appendiculatus* triggers abundance levels of known metabolites involved in metabolomic processes

**Fig. 4:**
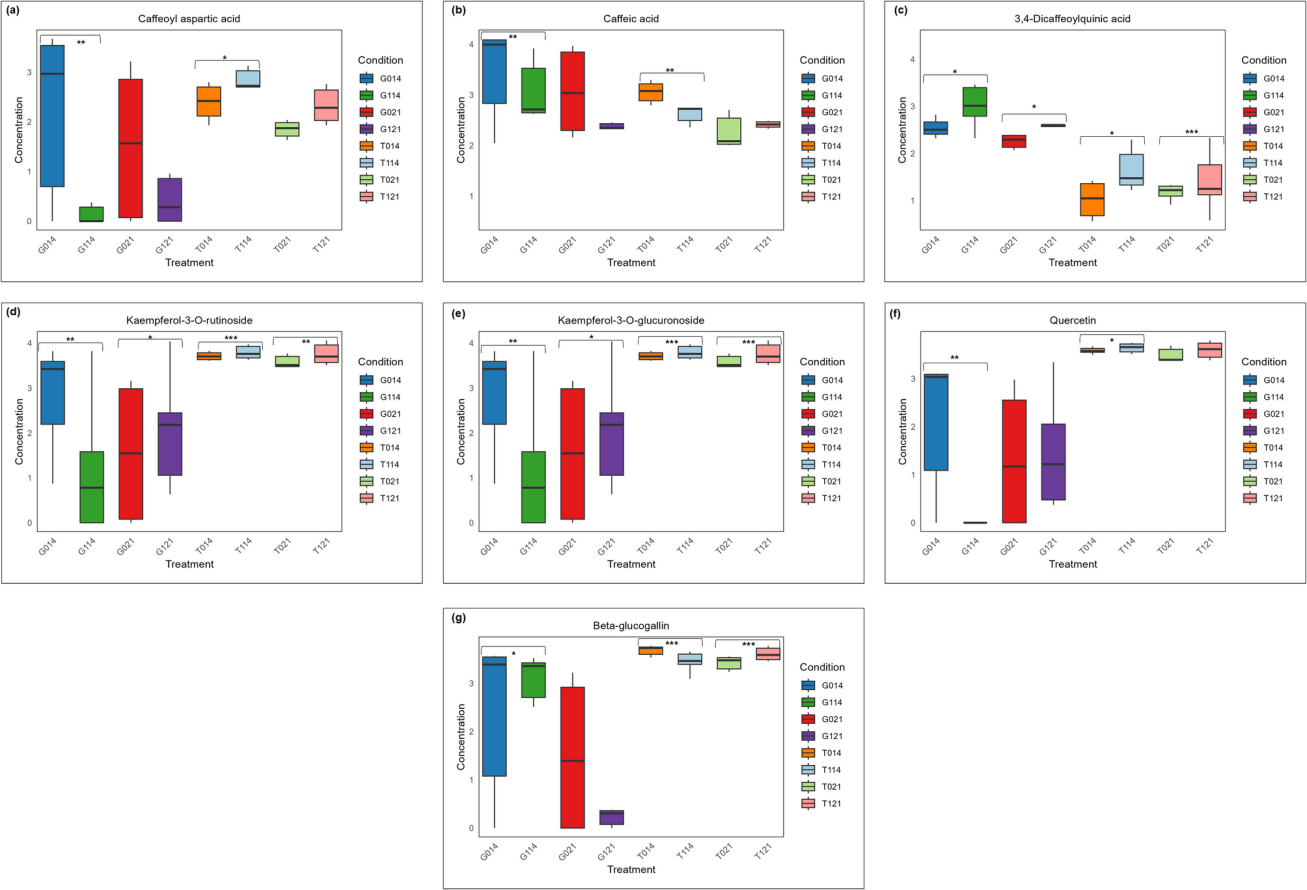
Box plots of metabolite abundance in *P.vulgaris* leaves at 14-dpi showing concentrations of (**a**) Caffeoyl aspartic acid, (**b**) Caffeic acid, (**c**) 3,4-Dicaffeoylquinic acid, (**d**) Kaempferol-3-O-rutinoside, (**e**) Kaempferol-3-O-glucuronoside, (**f**) Quercetin and (**g**) Beta-glucogallangin across treatments. *P*-values: * 0.05 (*P* ≤ 0.05) significant, ** 0.05 (*P* ≤ 0.01) very significant, *** 0.05 (*P* ≤ 0.001) highly significant. Denotations on figure are G: Golden Gate Wax, T: Teebus-RR-1, 14dpi: 14 days post infection, 21dpi: 21 days post infection, 1 after variety symbol indicates (G or T) indicates race 31 − 1 and 0 indicates mock inoculated plants

Untargeted profiling of metabolites in leaf samples of Golden Gate Wax and Teebus-RR-1 evaluated at 14- and 21-dpi revealed several known metabolites. A total of 30 known metabolites belonging to several chemical classes were found in both varieties at varying expression levels and significance (Table S2; Fig. 4). The Principal Component Analysis (PCA) revealed clustering of sample treatments and time intervals, post infection with *U. appendiculatus* (Fig. S3a).The OPLS model clustered samples similarly to the PCA model (Fig. S3b), indicating variations in metabolite expression patterns between the two varieties.

Infection with *U. appendiculatus* resulted in the expression of phenylpropanoids, flavonoids and phenolic acids among other compound classes at varying concentration levels upon comparison of mock and *U. appendiculatus* inoculated plants (Fig. 4). Interestingly, while the omnibus *P*-value indicated significant overall differences among metabolites, subsequent pairwise treatment comparisons identified only a limited number of metabolites that were statistically significant (Table S2). Intriguingly, phenylpropanoids such as caffeoyl aspartic acid, caffeic acid and 3,4-dicaffeoylquinic acid were regulated differentially between varieties at 14-dpi. For instance, caffeoyl aspartic acid at 14-dpi was very significantly (*P* ≤ 0.01) expressed in Golden Gate Wax, while significant in Teebus-RR-1 (Fig. 4a). Notably, infection with *U. appendiculatus* in Golden Gate Wax resulted in the down-regulation of caffeoyl aspartic acid (-3 log2FC) while an up-regulation was observed in Teebus-RR-1 (1 log2FC; Table S2). On the other hand, caffeic acid was very significantly expressed in both varieties, with a similar log2FC regulation pattern (Fig. 4b). Furthermore, similar expression patterns of 3,4-dicaffeoylquinic acid were observed in both varieties (Fig. 4c and Table S2). Kaempferol-3-O-rutinoside was down regulated in Golden Gate Wax (-2 log2FC) and Teebus-RR-1 (-1 log2FC) at 14-dpi, while highly significant (*P* ≤ 0.001) in Teebus-RR-1 and very significant in Golden Gate Wax (Fig. 4d and Table S2). Similar expression patterns were observed for kaempferol-3-O-glucuronoside (Fig. 4e) and quercetin in both varieties at 14-dpi (Fig. 4f). Strikingly, beta-glucogallin was significantly (*P* ≤ 0.05) down-regulated by Golden Gate Wax, while highly significantly up-regulated by Teebus-RR-1 at 14-dpi (Fig. 4g and Table S2).

**Fig. 5:**
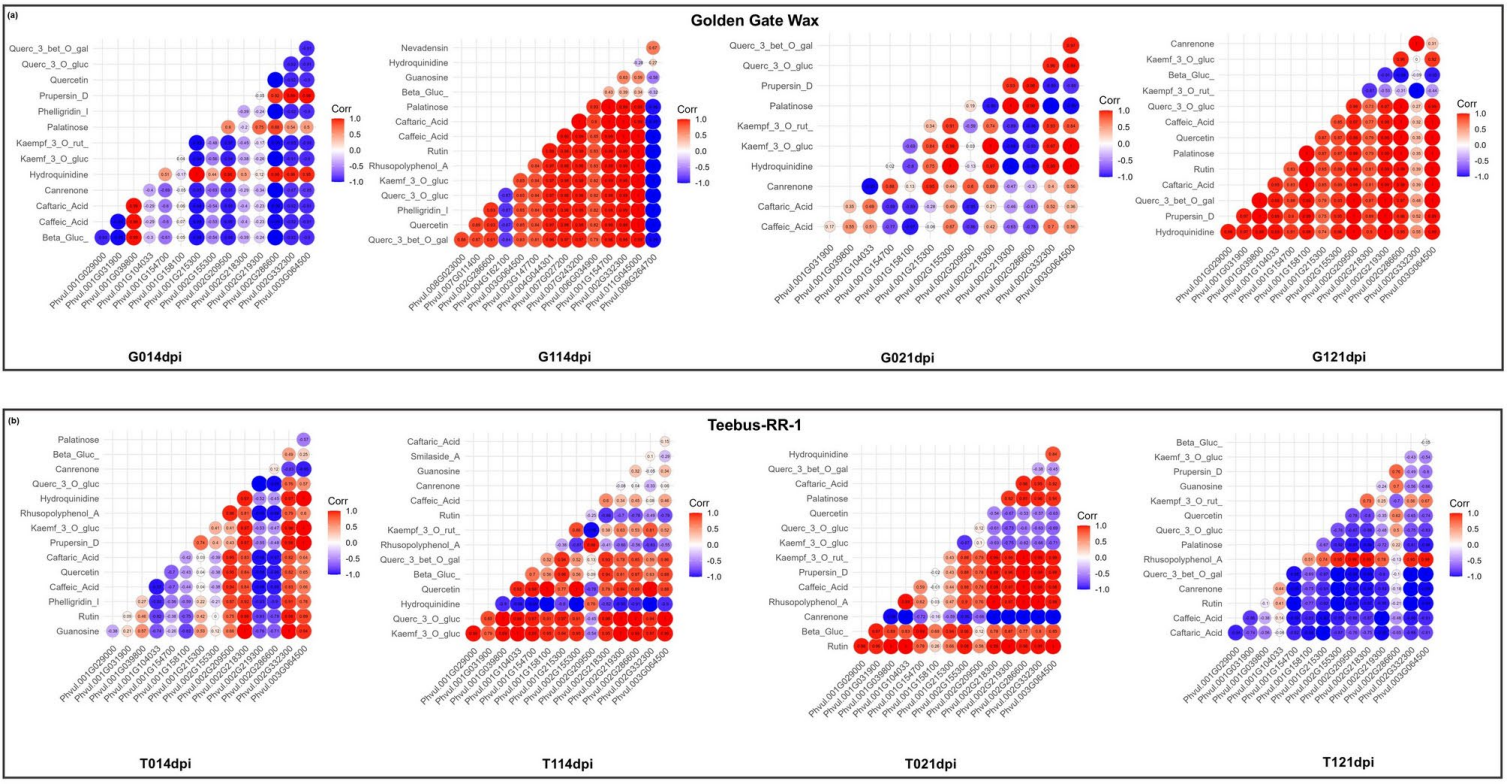
Correlation plots of metabolites and DEGs across treatments of Golden Gate Wax and Teebus-RR-1. (**a**) From left to right, Golden Gate Wax correlation plot of mock inoculated plants at 14-dpi (denoted as G014dpi), *U. appendiculatus* race 31 − 1 at 14-dpi (denoted as G114dpi), mock inoculated plants at 21-dpi (denoted as G021dpi), *U. appendiculatus* race 31 − 1 at 21-dpi (denoted as G121dpi). (**b**) From left to tight, Teebus-RR-1 correlation plot of mock inoculated plants at 14-dpi (denoted as T014dpi), *U. appendiculatus* race 31 − 1 at 14-dpi (denoted as T114dpi), mock inoculated plants at 21-dpi (denoted as T021dpi), *U. appendiculatus* race 31 − 1 at 21-dpi (denoted as T121dpi)

There was great variation or a “shift” in metabolite abundance in both varieties at 21-dpi. For example, there was no significant expression of caffeoyl aspartic acid (Fig. 4a) and caffeic acid (Fig. 4b) in both varieties at 21-dpi (Table S2). In addition, 3,4-dicaffeoylquinic acid was up-regulated at varying levels in both varieties and its expression was highly significant in Teebus-RR-1 (Fig. 4c). Similar regulation patterns of flavonoids kaempferol-3-O-rutinoside (Fig. 5d) and kaempferol-3-O-glucuronoside (Fig. 4e and Table S2) were observed in both varieties at 21-dpi. Interestingly, quercetin expression was not significant in both varieties at 21dpi (Fig. 5f and Table S2). On the other hand, beta-glucogallin was down-regulated in Golden Gate Wax while not significant and up-regulated by Teebus-RR-1 at highly significant expression levels (Fig. 4g; Table S2).

### Gene expression patterns triggered by *U. appendiculatus* in varieties of *P. vulgaris* with varying resistance to pathogen

The MGI DNBSEQ-G400 RNASeq instrument yielded over 90 million sequence reads with 90% of the reads mapping to the *P. vulgaris* v2.1 reference genome (Table S5). At 14-dpi, Golden Gate Wax had fewer genes that were not differentially expressed, thus having similar proportions of genes that were up- and down- regulated (Fig. S4a and c), while the opposite was observed at 21-dpi (Fig. S4b and c). Over 3000 genes were found to be differentially expressed (DEGs) between varieties across treatments (Fig. S4c). A total of 2806 DEGs were observed in Golden Gate Wax, while 369 DEGs were expressed in Teebus-RR-1 (Fig. S4c). Expression patterns at 14- and 21-dpi in Teebus-RR-1 were similar, with similar proportions of genes differentially expressed at each time point (Fig. S4c, e and f). Infection with *U. appendiculatus* triggered the differential expression of several genes to respond to the pathogen (Table S3 and S4). At 14-dpi, infection with *U. appendiculatus* revealed differential expression of several genes such as heat shock proteins (HSPs) and transcription factors (TFs) among others. Among these DEGs, 17.6 kDa class II heat shock protein (*HSP17.6II*: *Phvul.001G039800*), heat shock transcription factor A2 (*HSFA2*: *Phvul.009G078300*), winged-helix DNA-binding transcription factor family protein (*HSFB2B*: *Phvul.002G155300*) and heat shock transcription factor A6B (*HSFA6B*: *Phvul.001G154700*) were found to be expressed differentially upon *U. appendiculatus* infection. Interestingly, 2-oxoglutarate dehydrogenase, E1 component (*E1-OGDH2*: *Phvul.007G011400*), cold regulated gene 27 (*COR27*: *Phvul.001G022200*), galactinol synthase 2 (*GolS2*: *Phvul.007G203400*), lipases; hydrolases, acting on ester bonds (*RXW8*: *Phvul.001G031900*), osmotin 34 (*OSM34*: *Phvul.002G286600*), terpene synthase 21 (*TPS21*: *Phvul.011G143100*), terpene synthase 14 (*TPS14*: *Phvul.002G219300*), FKBP-type peptidyl-prolyl cis-trans isomerase family protein (*ROF2*: *Phvul.003G064500*) and rotamase FKBP 1 (*ROF1*: *Phvul.001G029000*) among others. Several genes expressed at 14-dpi were also observed at 21-dpi. For example, *GolS2*, *HSFA2*, *HSFB2B* and *HSFA6B* among others were expressed at varying expression levels. Distinctively, at 21-dpi, a gene that encodes a secreted peptide that enhances stress indued cell death (*BIA*: *Phvul.010G019701*) and WRKY DNA-binding protein 70 (*WRKY70*: *Phvul.008G081800*) were expressed differentially in both varieties at varying levels (Table S4). Targeted genes selected for qRT-PCR analysis were consistent with the expression patterns uncovered by RNA-Seq analysis (Fig. S5).

### Integrative analysis reveals a relationship between metabolites and genes expressed in *P. vulgaris*

Canonical correlation analysis revealed a negative correlation between metabolites and genes in mock inoculated plants of variety Golden Gate Wax at 14-dpi (Fig. 5a; G014dpi). For example, several metabolites such as caftaric acid, caffeic acid, kaempferol-3-O-glucuronoside and kaempferol-3-O-rutinoside among others were positively correlated to alpha/beta-Hydrolases superfamily protein (*MAGL4*: *Phvul.002G332300*), osmotin 34 and *ROF2* among others. Fascinatingly, quercetin 3-O-glucuronide, quercetin and quercetin-3-beta-O-galactoside among other metabolites in *U. appendiculatus* infected plants of Golden Gate Wax at 14dpi were negatively correlated with alpha-crystallin domain 32.1 (*ACD32.1*: *Phvul.008G264700*) and *MBF1C* (Fig. 5a; G114dpi). Teebus-RR-1 mock inoculated plants showed a positive correlation of rutin with *MAGL4*, Aldolase-type TIM barrel family protein (*HSA32*: *Phvul.002G218300*) and *MLP423* at 14-dpi (Fig. 5b; T014dpi). Metabolites kaempferol-3-O-glucuronoside, quercetin 3-O-glucuronide, quercetin and beta-glucogallin among others showed a positive correlation with genes *ROF2*, *MAGL4*, *OSM34* and *TPS14* among others at 14-dpi in Teebus-RR-1 *U. appendiculatus* infected plants (Fig. 5b; T114dpi).

Interestingly, a negative correlation of caffeic acid, caftaric acid and kaempferol-3-O-glucuronoside with *HSFA6B* (*Phvul.001G154700*) with was observed in Golden Gate Wax mock inoculated plants at 21-dpi (Fig. 5a; G021dpi). Infection with *U. appendiculatus* at 21-dpi induced a positive correlation of rutin, quercetin-3-beta-O-galactoside and caftaric acid with *HSFA6B* (Fig. 5a; G121dpi). In Teebus-RR-1 mock inoculated plants, the later gene was positive correlated to rutin, beta-glucogallin and caffeic acid (Fig. 5b; T021dpi). At 21-dpi, *U. appendiculatus* infection resulted in a negative correlation of the latter gene to rutin, caftaric acid, caffeic acid and quercetin 3-O-glucuronide (Fig. 5b; T121dpi). A similar correlation pattern can also be observed with *HSFB2B* with metabolites in both genotypes. Generally, Golden Gate Wax infected plants at 21-dpi had increased positive correlations between genes and metabolites, while the opposite was observed in Teebus-RR-1.

### Metabolomic and transcriptomic changes incited by *U. appendiculatus* include known biological pathways

**Fig. 6:**
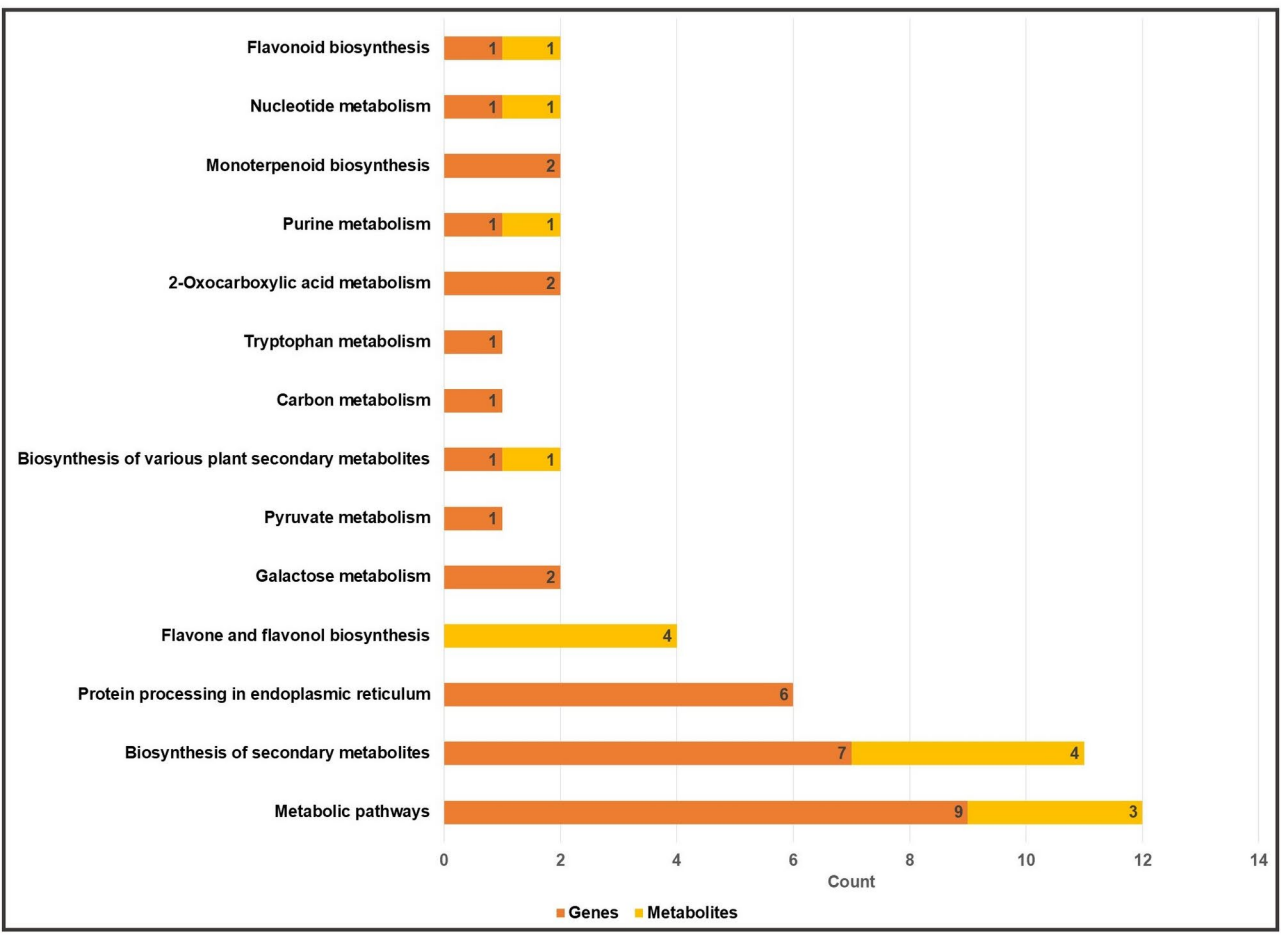
KEGG pathways enriched by genes and metabolites of *P. vulgaris* in response to *U. appendiculatus *infection

The impact of *U. appendiculatus* infection resulted in enrichment of known biological pathways including metabolic pathways, biosynthesis of metabolites, protein processing in the endoplasmic reticulum, carbon metabolism, monoterpenoid biosynthesis and purine metabolism among others (Fig. 6). Pathways were enriched by both metabolites and genes expressed in *P. vulgaris*. Only the expression levels of DEGs contributing to pathway enrichment were cluster heat mapped per treatment, alongside metabolite structures associated with those pathways (Fig. S6). For example, metabolic pathways were enriched by genes alpha/beta-hydrolases superfamily protein *MAGL4*, 2-isopropylmalate synthase 1 (*MAML-3*: *Phvul.001G104033*), *GolS1*, chloroplast beta-amylase (*CT-BMY*: *Phvul.003G226900*), O-methyltransferase family protein (*ASMT*: *Phvul.008G167800*), *TPS21* and *E1-OGDH2* (Fig. S6a). Intriguingly, the biosynthesis of secondary metabolites was enriched by *TPS14*, *MAML-3*, *CT-BMY*, *CYP82C4* (*Phvul.004G022000*), *ASMT* and *E1-OGDH2* (Fig. S6b). Furthermore, protein processing in the endoplasmic reticulum was majorly enriched by HSPs *ATHSP22.0*, *HSP17.6 C*, *HSP15.7* and *HSP17.8* together with calreticulin 3 (*PSL1*: *Phvul.011G053000*; Fig. S6c). Metabolites guanosine, rutin, quercetin and kaempferol-3-O-rutiniside contributed to enrichment of metabolomic pathways at varying expression levels (Fig. S6d).

## Discussions

Understanding the metabolites and genes involved in plant stress responses can inform strategies to optimize breeding programs, mitigating the exacerbated effects of pathogens and their impact on crop yields [45]. Studies on legumes such as chickpea and pea have demonstrated that flavonoids, glycerophospholipids, isoflavonoids, prenol lipids, polyphenols, terpenes, amino acids, carbohydrates, fatty acids carboxylic acids and derivatives among others contribute to fungal pathogen mediation [46, 47]. In a concurrent study, flavonoids, terpenoids, alkaloids and lipids were found to be incited by *U. appendiculatus* infections in Golden Gate Wax and Teebus-RR-1 [48]. By contrast, the impact of *U. appendciulatus* race 31 − 1 on common bean varieties is fragmentary, particularly the integration of metabolomic and transcriptomic tools to elucidate the pathogen’s biological impact on the crop. Susceptible variety, Golden Gate Wax revealed extensive metabolomic alterations at 14-dpi. In this study, *U. appendiculatus* altered the abundance levels of crucial such as such as, caffeoyl aspartic acid, caffeic acid, kaempferol-3-O-glucuronoside and quercetin derivatives among others.

For example, the down regulation of caffeoyl aspartic acid has been found to be a critical response strategy against *Botrytis cinerea* in wild grapevines [49]. Additionally, in tomato plants infected with *Fusarium oxysporum*, caffeoyl aspartic was found to inhibit fungal cell wall degradation [50]. Consistent with the latter studies, Golden Gate Wax exhibited a highly significant down-regulation of caffeoyl aspartic at 14-dpi, possibly implying weakened defense. This expression pattern in Golden Gate Wax can also be observed with beta-glucogallin, another defence associated metabolite, significantly down-regulated at 14-dpi. Beta-glucogallin is a hydrolysable tannin precursor that has been found to be involved in pant secondary metabolism [51]. Its role in secondary metabolism includes the biosynthesis of gallotannins and ellagitannins which serve as antimicrobial and antifeedant compounds against pathogens [52]. The regulation pattern of beta-glucogallin in response to fungal pathogens is not widely reported, therefore, more studies are crucial.

In this study, similar expression patterns of kaempferol-3-O-glucuronoside, kaempferol-3-O-rutinoside, and quercetin derivatives by both varieties at 14-dpi were observed, possibly indicating that these metabolites alone aren’t resistance determinants. Flavonoids are well documented as constitutive/basal defense metabolites that play a role in plant innate immunity [53]. In their basal defense, flavonoids are the first line of defence through their interaction with hormonal pathways salicylic acid (SA) and jasmonic acid (JA) [54]. 3,4-dicaffeoylquinic acid, a chlorogenic acid derivative, has been associated with enhanced disease resistance through multiple potential mechanisms, including antimicrobial activity, cell wall reinforcement, and modulation of defense-related signalling pathways [55]. In this study, 3,4-dicaffeoylquinic was expressed at highly significant levels in Teebus-RR-1, possibly indicating the plant’s ability to accumulate significant concentrations that conferred resistance. Interestingly, at 21-dpi, beta-glucogallangin was highly significantly up-regulated by Teebus-RR-1, while down-regulated and non-significant in Golden Gate Wax. This could indicate Golden Gate Wax’s inability to mount an effective pathogen response strategy by regulating critical metabolites for defence against *U. appendiculatus*. Despite similarities in regulation of some metabolites in both genotypes, metabolite expression and regulation patterns in Teebus-RR-1 were distinct. This may indicate the variety’s ability to recruit an assemblage of specific defence associated metabolites, and this may be observed through the variety’s expression pattern of metabolites such as caffeoyl aspartic acid and beta-glucogallangin among others.

Gene expression studies have uncovered several genes associated with plant immunity [56]. Major crops such as rice, maize, tomato and wheat have been extensively studied for specific gene families linked to plant-pathogen interaction [57–60]. However, transcriptome studies that extensively explore gene expression patterns of legumes under pathogenic stress remain limited. When plants are under pathogen attack, *HSPs* can intercept protein aggregation, cell translocation activities and degradation, particularly the stabilisation of protein re-folding [61, 62]. For example, in tomato, *HSP17.6.II* was found to be down-regulated by the plant in response to *Altenaria solani* [63]. Similar results were observed in Golden Gate Wax’s down-regulation of *HSP17.6.II*, meanwhile the opposite observed in Teebus-RR-1. The expression of *HSP17.8* in potato infected with *Phytophthora infestans* has been found to play a tole in plant immunity [64]. This is consistent with the results observed in Teebus-RR-1’s expression of *HSP17.8*. In pathogenic conditions, the activation of RLKs, especially those with leucine-rich repeats and intracellular kinase domains are key regulators of immune response and pathogen stress signalling [65]. *CRK29*, up-regulated in Golden Gate Wax at 14-dpi has been found to be associated with amplification of Reactive Oxygen Species (ROS) and localised cell death under pathogen stress conditions [66]. The induction of *CRK29* following *U. appendiculatus* infection at 14-dpi may have been delayed, permitting pathogen proliferation and subsequent systemic spread to secondary leaves. Notably, the induction of *Cytochrome P450* (*CYP*) monooxygenases during pathogen infection has been found to contribute to metabolite diversity, photosynthesis and photo-protection, which are critical for plant growth [67]. Furthermore, the synthesis of terpenoids, alkaloids and lipids among other metabolites that play a role in plant-pathogen interaction has also been linked to cytochrome genes [68]. In this study, for example, *CYP82C4* was down-regulated in Teebus-RR-1 while up-regulated in Golden Gate Wax. This occurrence in *Arabidopsis* mutant plants was found to be associated with iron deficiency and a positive correlation with other genes that aid in early stress response [69]. Though the current study did not involve mutant plants, we hypothesise that the up-regulation of *CYP82C4* by Golden Gate Wax at 14-dpi may indicate that there was increased iron accumulation on leaves, contributing to feeding the *U. appendciulatus* pathogen. Liu et al., [70] also elaborated that plants under pathogen attack tend to withhold iron to limit pathogen feeding and spread. Therefore, validation would require comparative evaluation using different varieties, time courses, strains and gene knockouts among other experimental approaches. Golden Gate Wax higher expression of terpene synthase 14 (*TPS14*) at 14-dpi in comparison to Teebus-RR-1, which may correlate with enhanced methyl jasmonate (MeJA) biosynthesis. Previous studies have associated elevated MeJA levels with reduced plant growth and premature defoliation [71, 72]. At 21-dpi, *WRKY70* was down-regulated in Teebus-RR-1 and generally, *WRKY* genes are widely studied for their contribution to pathogen triggered immunity (PTI) and effector triggered immunity (ETI) to plant pathogens [73]. In chickpea infected with *Fusarium oxysporum*, increased expression of *WRKY70* was associated with pathogen susceptibility [74]. This finding is consistent with the expression patterns of *WRKY70* by Golden Gate Wax.

In this study, the use of correlation was aimed at gaining insights on how genes interact with metabolites upon *U. appendiculatus* infection. *ACD32.1* in Golden Gate Wax at 14-dpi was found to be negatively correlated to several metabolites. There is limited information on the role of *ACD32.1* in plant-pathogen interaction. In a study evaluating temperature resistance of cassava [75] and *P. vulgaris* [76], *ACD32.1* was found to be expressed positively as a response strategy. The inverse relationship of *ACD32.1* with metabolites in this instance could reflect suboptimal coordination of molecular defense against *U. appendiculatus*. On the other hand, the observed inverse correlation between *ROF2* expression and levels of caftaric acid, caffeic acid, kaempferol-3-glucuronoside, and kaempferol-3-O-rutinoside suggests a potential regulatory role for *ROF2* in suppressing these metabolites. *ROF2* like *ROF1* has been found to participate in long term thermo-resistance in plants [77], though further validation is needed to establish causal relationships between *ROF2* and phenolic compound biosynthesis. Interestingly, *OSM34* has been found to be a responsive gene against bacterial and fungal pathogens through its up-regulation upon pathogen perception [78]. In a study evaluating the efficacy of *Trichorderma velutinum* in controlling *Rhizoctonia solani* in common bean, increased levels of *OSM34* were found play a role in plant defence. In this study, *OSM34* was found to be positively correlated to number of metabolites, especially flavonoids at 14-dpi in infected plants of both varieties, thus indicating its involvement in the biosynthesis of secondary metabolites. Furthermore, in Teebus-RR-1, *TPS14* was negatively correlated to several metabolites at 14-dpi for mock inoculated plants, while positively correlated in *U. appendiculatus* infected plants. In a study evaluating soyabean’s resistance to anthracnose, intense adjustments of *TPS14* (a terpenoid backbone and plant hormone metabolism contributor) was found to improve soyabean resistance to anthracnose [79]. The interactive properties of *MAGL4*, *ROF2*, *OSM34*, and *TPS14* were similar, especially in mock inoculated conditions of Golden Gate Wax, mock and *U. appendiculatus* infected conditions of Teebus-RR-1 at 14-dpi. Based on these observations, we hypothesise that increased accumulation of these candidate genes relative to metabolite levels may contribute to pathogen recognition and innate immune responses. Further investigation of these gene-metabolite relationships could identify potential targets for improving resistance to *U. appendiculatus* in breeding programs.

Increased expression of *HSFB2B* in *Arabidopsis* has been found to be associated with pathogen resistance [80]. *HSFA6B* in wheat [81] and barley [82] has also been found to improve plant heat resistance through activation of other stress signalling genes [81]. In this study, DEGs *HSFB2B* and *HSFA6B* were positively correlated to several metabolites in Golden Gate Wax and Teebus-RR-1 in *U. appendiculatus* infected plants at 21-dpi, thus signalling the role of these genes in plant-pathogen interaction. However, it should also be noted that for varieties to confer resistance to plant pathogens, gene expression synchronisation and timing of biological processes that are relevant for plant immunity is important [83]. The observed positive metabolite-gene correlations in Golden Gate Wax could suggest potential resource allocation trade-offs, possibly linked to its increased susceptibility [84]. Meanwhile, Teebus-RR-1 showed stronger negative interactions at 21-dpi, along with more coordinated expression patterns of specific genes such as *MAGL4*, *ROF2*, *OSM34*, and *TPS14* with subsets of flavonoid/phenylpropanoids metabolites across time points. While this observation has been found to play a role in plant-pathogen resistance and overall plant development [85], further mechanistic studies are needed to validate these associations.

Metabolic pathways play a role in the biosynthesis of key specialised metabolites and genes that are responsible for key functions that regulate plant metabolic flux [86]. For example, at 14-dpi *E1-OGDH2* was down-regulated in Golden Gate Wax, while up-regulated in Teebus-RR-1 when comparing expression concentrations in untreated versus *U. appendiculatus* treated samples (Fig. S6a). Similar expression patterns in *Arabidopsis* were found to decrease respiration rates, thus impacting Carbon-N metabolism [87]. Studies on the functions of *E1-OGDH2* under pathogen stress in legumes are required to provide understanding on the regulation patterns of the gene. The biosynthesis of secondary metabolites during pathogen infection plays a role in protecting plants from pathogen invasion by promoting plant growth and development [88]. A role player in the biosynthesis of secondary metabolites is *TPS14* (Fig. S6b) which has been found to play a role in terpenoid biosynthesis, important for ABA (abscisic acid biosynthesis) [79, 89]. In our findings, *TPS14* was evident in both varieties at the two time points, signalling the role played by the gene in plant-pathogen interaction. Protein processing in endoplasmic reticulum was majorly driven by the expression of *HSPs* (Fig. S6c). *ATHSP22.0* and *HSP17.8* were notably involved in the regulation of protein processing in endoplasmic reticulum. Pathogen infection hijacks the endoplasmic reticulum, triggering the unfolded protein response (UPR) that can initiate programmed cell death (PCD) under severe stress conditions [90]. While HSPs are well characterised thermal stress response genes, their role in plant-pathogen interactions remains poorly understood. Notably, in heat-stressed grapevines, *ATHSP22.0* was shown to modulate endoplasmic reticulum protein processing [91], suggesting potential crosstalk between HSP-mediated protein folding and pathogen-induced ER stress responses. In this study, Golden Gate Wax at 14-dpi regulated *HSPs* inconsistently and this was observed from the varying regulation patterns of numerous genes. An example is the excessive down regulation of *HSP15.7* at 14-dpi, which may have potentially exacerbated stress in the endoplasmic reticulum [92]. The expression of metabolites during pathogen stress occurrences is important in plant-pathogen interaction as metabolites are activators of stress resistant genes [93]. Defence related metabolites (Fig. S6d) and genes are interconnected and collectively improve plant-pathogen resistance by modulating key signalling pathways including MAPK signalling, phytohormone crosstalk, transcriptome reprogramming and biosynthesises of secondary metabolites among others [53–55, 94].

## Conclusion

This study presents integration of metabolomics with transcriptomics to gain insights on the response of common bean to rust infection at important plant growth stages. Results obtained from this study revealed a linkage between metabolites and genes expressed in common bean. Inclusively, these findings epitomize the strength of integrating metabolomics with transcriptomics to give in-depth analysis of biological products that are triggered by stressors, especially fungal pathogens. In addition, the results of this study revealed metabolite and gene expression trends among susceptible and resistant varieties to rust infection. Timely expression of specific metabolites and genes optimally upon pathogen attack is crucial in plant-pathogen interaction. These findings provide a basis for future studies aimed at targeted breeding techniques to accelerate rust resistance in common bean. This study only utilised two varieties (susceptible and resistant) to evaluate their response to rust race 31− 1 at two critical time points under controlled conditions. Further work should be conducted with inclusion of more varieties, races/cocktail of rust and time points to advance metabolome and transcriptome knowledge. In addition, advances to this study can also include the use of metabolite and gene knockout techniques to observe plant-pathogen interactions. The results and methodologies of this study can be used as a basis for common bean rust resistance breeding.

## Electronic supplementary material

Below is the link to the electronic supplementary material.

Supplementary Material 1

Supplementary Material 2

Supplementary Material 3

Supplementary Material 4

Supplementary Material 5

Supplementary Material 6

Supplementary Material 7

Supplementary Material 8

Supplementary Material 9

Supplementary Material 10

Supplementary Material 11

## Data Availability

Metabolomics data presented in this study has been deposited in the MetaboLights repository (MTBLS6972). The sequence raw reads that were generated from this study have been submitted to the National Centre for Biotechnology Information (NCBI) SRA with project ID: PRJNA1061833.
