# Supplementary figures and images for: Exploring associations between metabolites and gene transcripts of common bean (Phaseolus vulgaris L.) in response to rust (Uromyces appendiculatus) infection
Source: BMC Plant Biol. 2025 May 1;25:568. doi: 10.1186/s12870-025-06584-w (PMC12044953; doi:10.1186/s12870-025-06584-w)

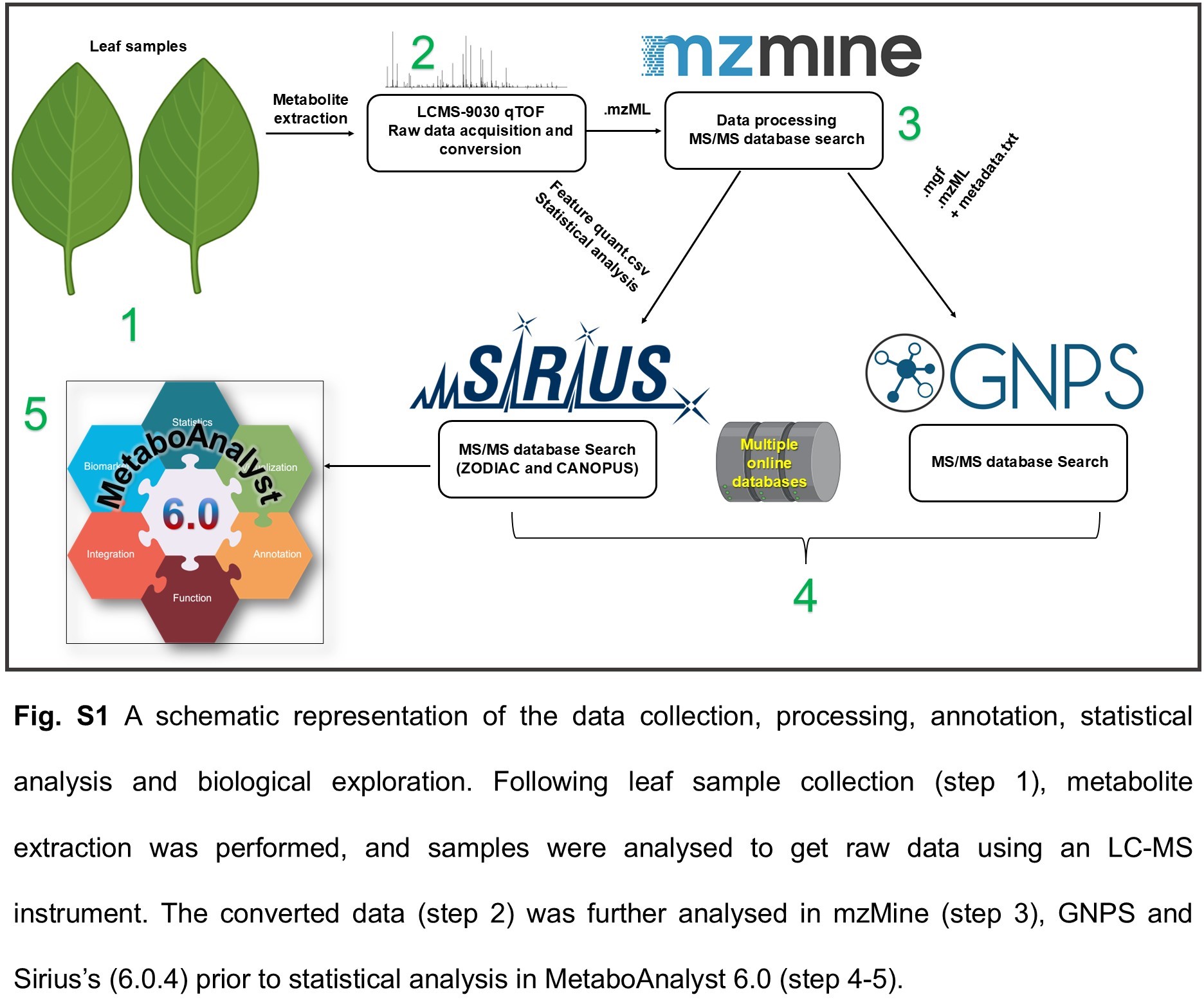

Supplement: Supplementary file 1 — Supplementary Material 1 [file 12870_2025_6584_MOESM1_ESM.jpg]

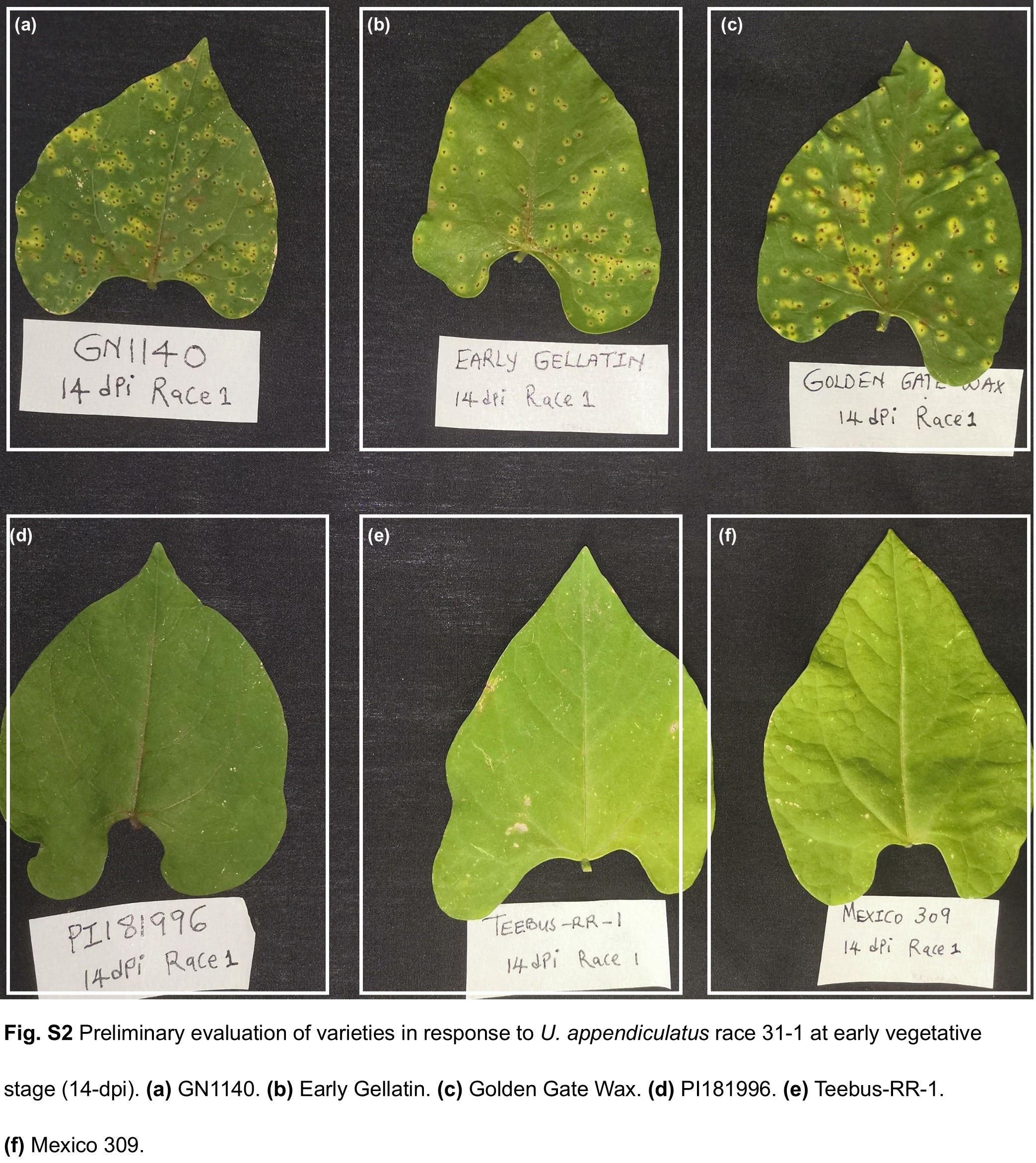

Supplement: Supplementary file 2 — Supplementary Material 2 [file 12870_2025_6584_MOESM2_ESM.jpg]

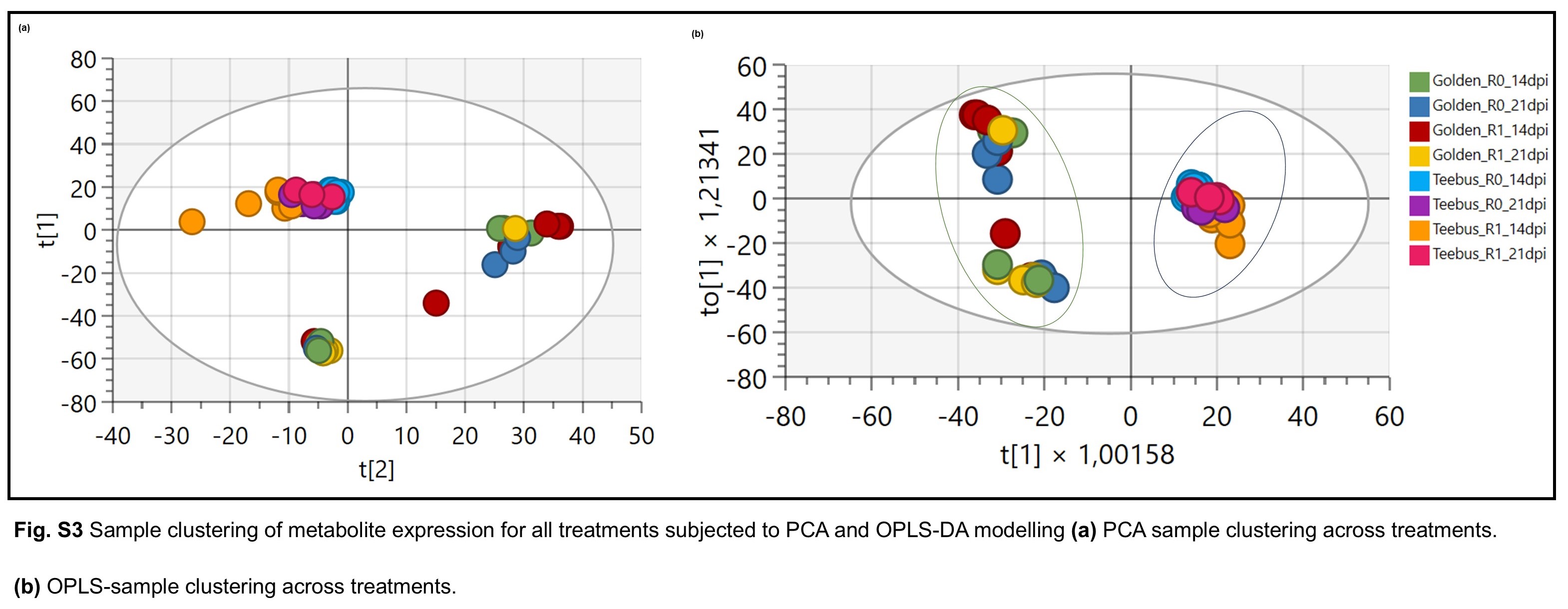

Supplement: Supplementary file 3 — Supplementary Material 3 [file 12870_2025_6584_MOESM3_ESM.jpg]

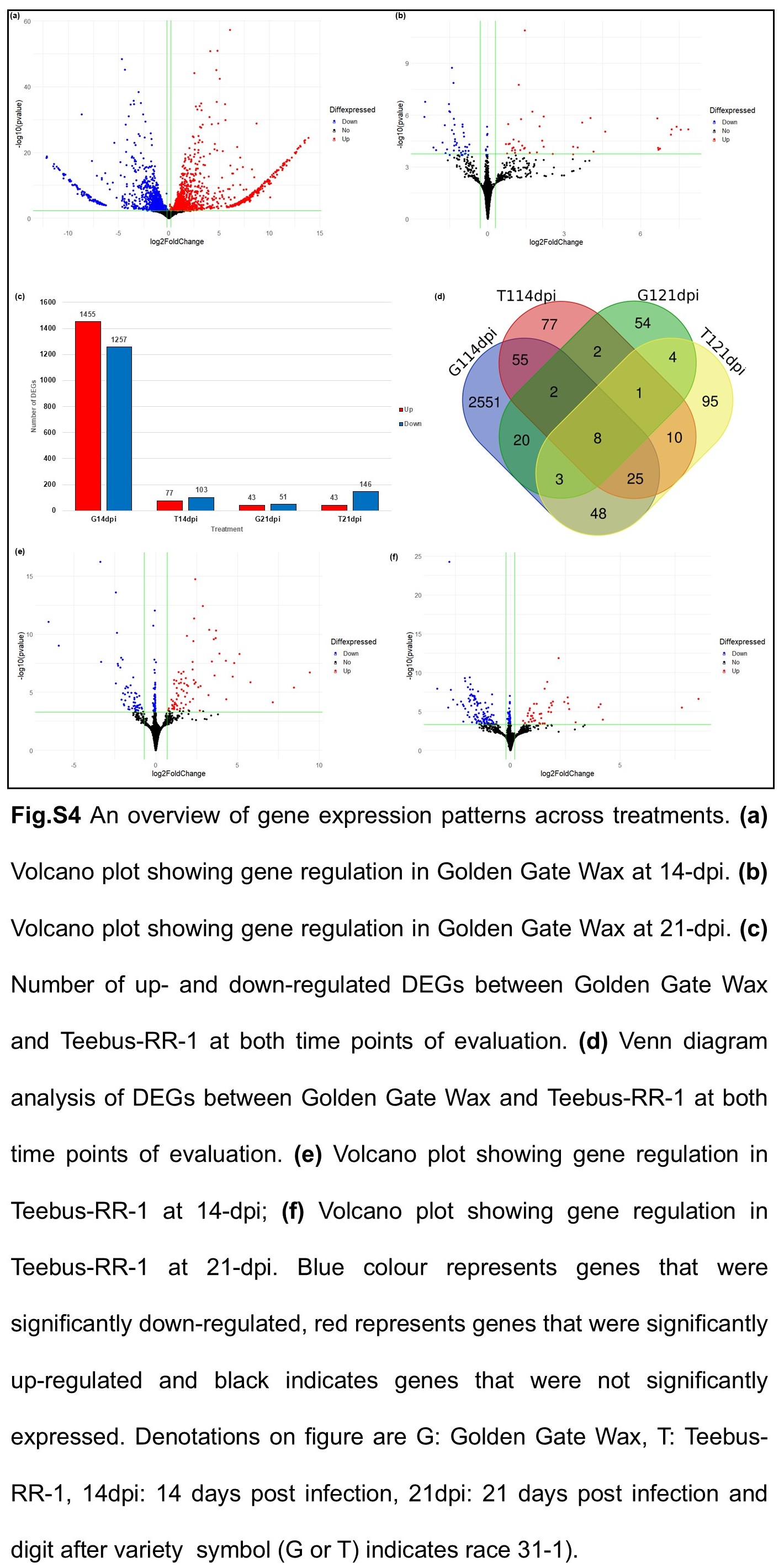

Supplement: Supplementary file 4 — Supplementary Material 4 [file 12870_2025_6584_MOESM4_ESM.jpg]

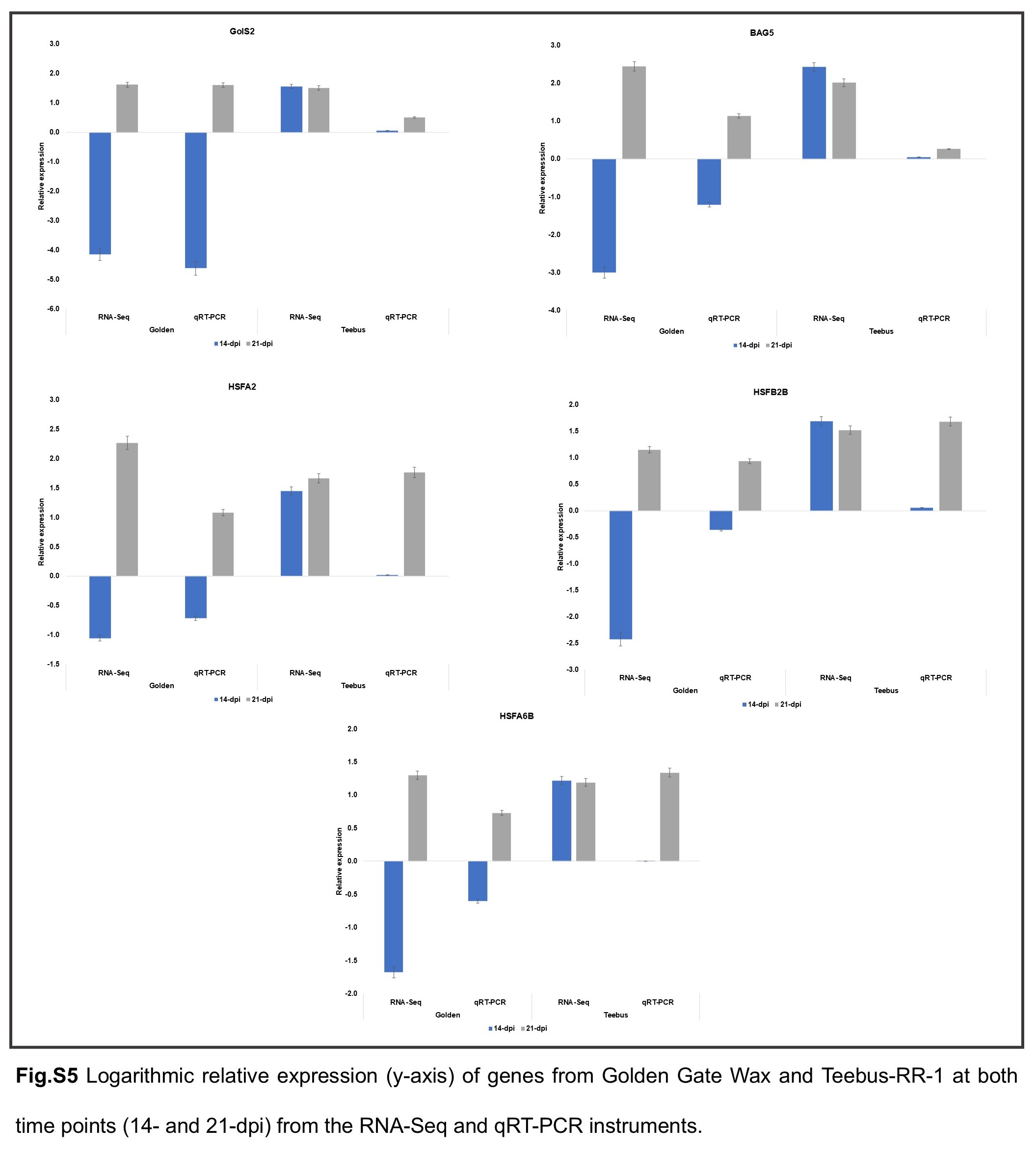

Supplement: Supplementary file 5 — Supplementary Material 5 [file 12870_2025_6584_MOESM5_ESM.jpg]

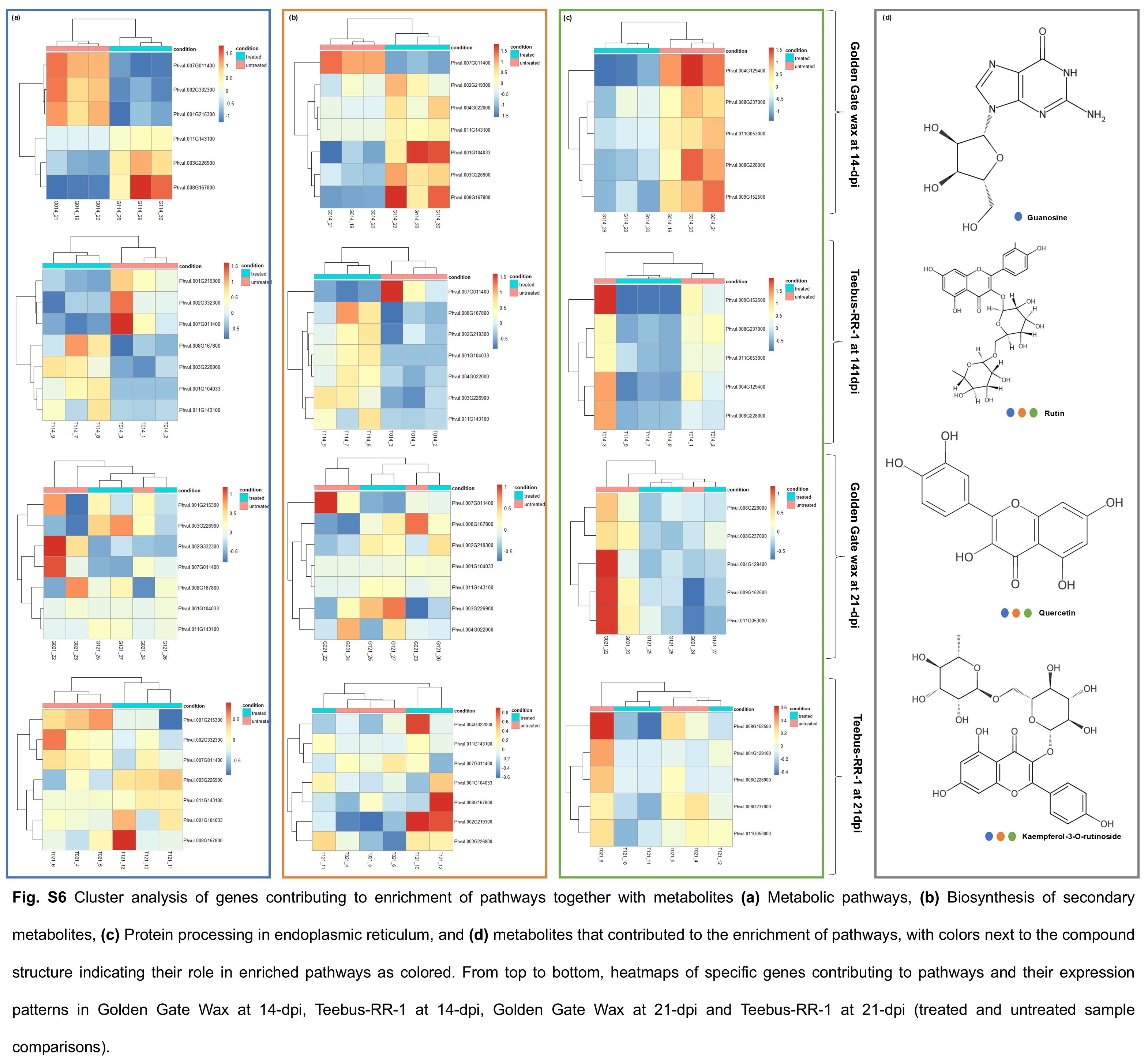

Supplement: Supplementary file 6 — Supplementary Material 6 [file 12870_2025_6584_MOESM6_ESM.jpg]
